# Knee dGEMRIC at 7 T: comparison against 1.5 T and evaluation of T_1_-mapping methods

**DOI:** 10.1186/s12891-018-2071-1

**Published:** 2018-05-16

**Authors:** Pernilla Peterson, Carl Johan Tiderius, Emma Olsson, Björn Lundin, Lars E. Olsson, Jonas Svensson

**Affiliations:** 1 Medical Radiation Physics, Department of Translational Medicine, Lund University, Inga Marie Nilssons gata 49, SE-205 02 Malmö, Sweden; 2 Department of Oncology and Radiation Physics, Skåne University Hospital, Inga Marie Nilssons gata 49, SE-205 02 Malmö, Sweden; 3 Orthopedics, Department of Clinical Sciences, Lund University, Skåne University Hospital, SE-221 85 Lund, Sweden; 4 Department of Medical Imaging and Physiology, Skåne University Hospital, SE-221 85 Lund, Sweden

**Keywords:** dGEMRIC, Cartilage, 7 T, Inversion recovery, Variable flip angle, Look-locker

## Abstract

**Background:**

dGEMRIC (delayed Gadolinium Enhanced Magnetic Resonance Image of Cartilage) is a well-established technique for cartilage quality assessment in osteoarthritis at clinical field strengths. The method is robust, but requires injection of contrast agent and a cumbersome examination procedure. New non-contrast-agent-based techniques for cartilage quality assessment are currently being developed at 7 T. However, dGEMRIC remains an important reference technique during this development. The aim of this work was to compare T_1_ mapping for dGEMRIC at 7 T and 1.5 T, and to evaluate three T_1_-mapping methods at 7 T.

**Methods:**

The knee of 10 healthy volunteers and 9 patients with early signs of cartilage degradation were examined at 1.5 T and 7 T after a single (one) contrast agent injection (Gd-(DTPA)^2−^). Inversion recovery (IR) sequences were acquired at both field strengths, and at 7 T variable flip angle (VFA) and Look-Locker (LL) sequences were additionally acquired. T_1_ maps were calculated and average T_1_ values were estimated within superficial and deep regions-of-interest (ROIs) in the lateral and medial condyles, respectively.

**Results:**

T_1_ values were 1.8 (1.4–2.3) times longer at 7 T. A strong correlation was detected between 1.5 T and 7 T T_1_ values (*r* = 0.80). For IR, an additional inversion time was required to avoid underestimation (bias±limits of agreement − 127 ± 234 ms) due to the longer T_1_ values at 7 T. Out of the two 3D sequences tested, LL resulted in more accurate and precise T_1_ estimation compared to VFA (average bias±limits of agreement LL: 12 ± 202 ms compared to VFA: 25 ± 622 ms). For both, B_1_ correction improved agreement to IR.

**Conclusion:**

With an adapted sampling scheme, dGEMRIC T_1_ mapping is feasible at 7 T and correlates well to 1.5 T. If 3D is to be used for T_1_ mapping of the knee at 7 T, LL is preferred and VFA is not recommended. For VFA and LL, B_1_ correction is necessary for accurate T_1_ estimation.

## Background

Osteoarthritis is a common, painful, and disabling condition characterized by degradation and loss of cartilage. Although the disease progresses slowly, early detection is critical for development of treatment strategies which may prevent or slow down degradation before the cartilage is irreversibly lost.

The delayed Gadolinium Enhanced Magnetic Resonance Imaging of Cartilage (dGEMRIC) technique is a well-established method for early assessment of cartilage quality in osteoarthritis [1]. Using this technique, the distribution of Gd-(DTPA)^2−^ contrast agent in cartilage after intravenous injection is assessed with quantitative T_1_ mapping. The estimated T_1_ is assumed to be indirectly related to the content of glycosaminoglycan (GAG) which is known to decrease early in osteoarthritis. The method is robust and has proved to sensitively detect early degenerative cartilage processes [2, 3] and loss of cartilage quality [4]. However, the technique requires injection of contrast agent, which in addition to a cumbersome examination procedure may also lead to long-term gadolinium deposits [5]. Thus, current development of magnetic resonance imaging (MRI) methods for assessment of cartilage quality is focused on methods that do not require contrast agent injection (e.g. GAG Chemical Exchange Saturation Transfer (gagCEST), ^23^Na imaging, T_2_ mapping, and T_1ρ_ mapping [6]). In the development process of these new techniques there is still a real need for an established method for cartilage quality evaluation to use as a reference. For this purpose dGEMRIC may still be the most suitable choice.

gagCEST and ^23^Na imaging benefit from the use of an ultra-high field strength, such as 7 T [7, 8]. Most dGEMRIC studies have so far been conducted at clinical field strengths. To enable the use of dGEMRIC as a reference tool during the development of the new techniques, there is a need to first validate dGEMRIC also at 7 T.

Translating the dGEMRIC technique to an ultra-high field strength may have some advantages but there are also several challenges. Increasing the field strength increases the signal-to-noise ratio (SNR), which may be used to improve either the measurement precision or imaging resolution. However, a higher field strength also increases the expected T_1_ values [9] and decreases the relaxivity of Gd-(DTPA)^2−^ [10]. These effects may require an altered dGEMRIC protocol and could reduce the sensitivity of the dGEMRIC experiment.

T_1_ mapping is a core component of the dGEMRIC technique and several methods have been suggested in the literature. The gold standard approach is the 2D inversion recovery (IR) technique, but also 3D approaches such as the variable flip angle (VFA) [11] and Look-Locker (LL) techniques have been increasingly used over the last years [12–14]. Several challenges for accurate T_1_ measurements are expected when moving to a higher field strength. First, the longer T_1_ values likely require longer inversion and repetition times which increases the acquisition time. Second, the B_1_ field is likely more inhomogeneous at ultra-high field strengths compared to clinical field strengths. This may affect the quality of the inversion pulse for the IR and LL experiments, but may also make B_1_ correction approaches necessary for the VFA and LL techniques [14, 15]. For dGEMRIC at 7 T, IR [16] and VFA [17, 18] have previously been used for T_1_ mapping, but as no quantitative comparison between the methods has been performed, further investigation is needed to find the optimal T_1_-mapping approach at ultra-high field strength.

The aim of this study was to evaluate the feasibility of T1 mapping for knee dGEMRIC at 7 T by comparison against 1.5 T in human subjects in vivo. In order to identify a preferred choice of T_1_-mapping approach at the ultra-high field strength, we additionally aim to compare and evaluate three T_1_-mapping techniques – IR, VFA, and LL.

## Methods

### Human subjects

The study was approved by the regional ethical review board and all human subjects gave their written informed consent. To increase the expected range of T_1_ values, both healthy volunteers (*N* = 10; 6 males, 4 females; median (range) age = 33.5 (23–56) years; body mass index (BMI) = 23.6 (20.7–26.3) kg/m^2^) and patients with early degenerative changes in the knee cartilage (*N* = 9; 6 males, 3 females; median (range) age = 42.9 (36–48) years; BMI = 30.1 (23.8–33.3) kg/m^2^) were included in the study. The inclusion criterion for the healthy volunteers was: No previous history of pain or other problem with the knee to be examined. Inclusion criteria for the patients were: superficial degenerative cartilage changes on the medial femoral condyle but no significant cartilage loss or fissuring deeper than 50% of the cartilage thickness as verified by arthroscopy conducted no more than 5 years before the MRI. The median time between arthroscopy and and imaging for the included subjects was 2.4 years (min 1.0 and max 2.8 years). Exclusion criteria for all subjects were: Kidney disease and implants which were not MRI compatible or risked induce artifacts.

### Experiment procedure

Upon arrival at the hospital, an intravenous injection of a double dose (0.2 mmol/kg body weight) of Gd-(DTPA)^2−^ (Magnevist®, Bayer Schering Pharma AG, Berlin, Germany) was administered. The subjects were then asked to walk at an easy pace along a specified path during 10 min to help distribution of the contrast agent in the cartilage [19].

Either the left or right knee of all subjects were imaged using both 1.5 T and 7 T MRI scanners (Philips Achieva dStream and Philips Achieva AS, Best, the Netherlands). Both examinations were conducted during one session after the same contrast agent injection. Half of the healthy subjects were examined at 1.5 T first and half at 7 T first. The order of the patient examinations was determined by practical scheduling considerations. Start of the first imaging session was planned such that the first IR sequence (see details below) was initiated 120 min after the contrast agent injection. The order of the sequences in the scan protocols was planned to ensure a minimum delay between the acquisitions of the IR sequences at the two field strengths. The subjects were transported between the two scanner rooms sitting in a wheel chair to minimize redistribution of the contrast agent in the knee joint between the examinations.

### MRI examination

During the examinations, the knee was immobilized slightly bent in dedicated knee coils (1.5 T: receive only dStream Knee 15ch Coil, 7 T: transmit and receive QED Knee Coil 1TX / 28RX) using pads. A series of IR sequences with different inversion times (TI) were acquired at both 1.5 T and at 7 T. 2D slices were centered over the medial and lateral condyle, respectively, and imaged in separate sequences (single slice). At 1.5 T, 6 TIs were acquired (TI = 50 ms, 100 ms, 200 ms, 400 ms, 800 ms, and 1600 ms). Other parameters were: repetition time (TR) = 2000 ms, echo time (TE) = 7 ms, field of view (FOV) = 120x120x3 mm^3^, bandwidth = 402 Hz/pixel, echo train length = 11, matrix size = 256 × 256, and acquisition time (TA)/IR sequence = 46 s. The corresponding parameters for the 7 IR acquisitions at 7 T were TI = 50 ms, 100 ms, 200 ms, 400 ms, 800 ms, 1600 ms, and 3800 ms, TR = 4000 ms, TE = 7 ms, FOV = 120x120x3 mm^3^, bandwidth = 338 Hz/pixel, echo train length = 11, matrix size = 256 × 256, and TA/IR sequence = 1 min and 36 s. At 1.5 T a short diagnostic protocol was also executed in addition to the IR acquisition for all subjects. This was later used to exclude unexpected pathology and to aid in determining that the cartilage had adequate thickness for ROI evaluation.

At 7 T two different 3D T_1_ methods were additionally evaluated: VFA and LL. For VFA, two 3D gradient echo sequences covering the knee joint were acquired with a non-selective excitation pulse and flip angles = 7° and 39°, TR = 30 ms, TE = 2.7 ms, FOV = 120 × 120 mm^2^, slice thickness = 3 mm, pixel bandwidth = 338 Hz, matrix size = 256 × 256, and TA/sequence = 4–6 min depending on number of slices. The flip angles were optimized expecting a T_1_ of 700 ms [20]. For LL, a 3D gradient echo sequence was acquired with flip angle = 6°, TR = 5000 ms, time between each excitation pulse 5.5 ms, TE = 2.7 ms, FOV = 140x140x3 mm^3^, pixel bandwidth = 338 Hz, echo train length = 15, matrix size = 256 × 256, and TA = 13–15 min depending on number of slices. 24 inversion times were acquired ranging from 16 ms – 3466 ms.

Finally, a Dual Refocusing Echo Acquisition Mode (DREAM) method for B_1_ mapping [21] was acquired at 7 T with: flip angle = 15°, TR = 5.7 ms, TE = 2.9 ms, FOV = 120 × 120 mm^2^, slice thickness = 3 mm, pixel bandwidth = 1695 Hz, matrix size = 120 × 110, and TA = 2–3 min depending on number of slices.

The acquisition of the IR sequences were prioritized and were acquired in all subjects. In some cases the 7 T examinations were limited by time, and for this reason both 3D sequences where not acquired in all subjects. VFA was acquired in 10 subjects (5 patients and 5 healthy subjects) and LL in 14 subjects (5 patients and 9 healthy subjects). The total scan time for each volunteer was approximately 20 min at 1.5 T and 50 min at 7 T.

### Estimation of T_1_ maps

Voxel-based T_1_ maps were created using the data from the three different methods (IR, VFA, and LL) in home-written Matlab scripts (v. R2013b, Mathworks, Nattick, USA). When necessary, affine image registration using the imregister Matlab function was conducted between the various image sequences before further T_1_ estimation. The T1 calculations in the scripts were validated with phantom experiments using Ni-doped agarores gel phantoms with known T1 relaxation times before the start of this study (data not shown). The following calculations were performed:

#### B_1_ error estimation

The relative B_1_ error (*c*), expressed as a fraction of the nominal flip angle, was mapped using the DREAM sequence as described above [21]. An average value (*c*_*ROI*_) within the investigated region-of-interest (ROI) (see below) was estimated and used for correction of VFA and LL data.

#### IR

T_1_ was estimated with a 3-parameter fit to Eq. (1) using a Levenberg-Marquardt non-linear-least-squares algorithm:

1 $$ {S}_{TI}={S}_0\left(1-k{e}^{-\frac{TI}{T_1}}+{e}^{-\frac{TR}{T_1}}\right) $$

*S*_*TI*_ is the signal acquired at inversion time *TI*, *S*_*0*_ is the estimated signal at *TI* = 0, and *k* is the quality of the inversion pulse. A perfect inversion pulse corresponds to *k* = 2.

For estimation of T_1_ at 1.5 T, all six acquired *S*_*TI*_ were used. For 7 T, T_1_ was estimated both from the first six *S*_*TI*_ and from all seven *S*_*TI*_s to investigate the importance of the additional longer TI at 7 T.

#### LL

From LL data (*S*_*TI*_) the apparent T_1_ (T_1_*), *M*_*A*_, and *M*_*B*_ were estimated in a 3-parameter fit to the following equation using a Levenberg-Marquardt non-linear-least-squares algorithm [22]:

2 $$ {S}_{TI}={M}_A-{M}_B{e}^{-\frac{TI}{T_1\ast }} $$

For estimation of the actual T_1_ the following equation was used:

3 $$ {T}_1=\frac{1}{\frac{1}{T_1\ast }+\frac{\ln \left(\cos \left( c\alpha \right)\right)}{TR}} $$

The nominal flip angle is represented by *α*, and the relative error of the flip angle is given by the factor *c*. Both B_1_-uncorrected (*c* = 1) and B_1_-corrected (*c* = *c*_*ROI*_, see above) T_1_ values were estimated for comparison.

#### VFA

The T_1_ was estimated from the signals *S*_*1*_ and *S*_*2*_ acquired at the two flip angles *α*_*1*_ and *α*_*2*_ according to [23]:

4 $$ {T}_1=\frac{TR}{\log \left(\frac{\sin \left(c{\alpha}_1\right)\cos \left(c{\alpha}_2\right)-\frac{S_1}{S_2}\sin \left(c{\alpha}_2\right)\cos \left(c{\alpha}_1\right)}{\sin \left(c{\alpha}_1\right)-\frac{S_1}{S_2}\sin \left(c{\alpha}_2\right)}\right)} $$

A B_1_-uncorrected T_1_ was obtained by setting *c* = 1, whereas *c* = *c*_*ROI*_ was used for a B_1_-corrected T_1_.

### Data analysis

Data analysis and ROI definition was performed in Matlab (v. R2013b, Mathworks, Nattick, USA). Two ROIs (one superficial and one deep) were drawn in each of the load-bearing lateral and medial femoral condyles for each field strength and method, respectively. Each ROI covered half the depth of the femoral cartilage from the center of the tibial plateau to the posterior boundary of the posterior meniscus. All ROIs were drawn by two readers to evaluate the variance in ROI definition. IR ROIs were drawn by Reader 1 and Reader 2 with 19 and 2 years of experience, respectively. VFA and LL ROIs were drawn by Reader 2 and Reader 3 (1 year of experience). For the 3D sequences, care was taken to choose the slice that best matched the position of the IR slice. In addition, the adjacent two slices were also evaluated for both 3D approaches to investigate the uncertainty introduced by non-identical slice positioning. To exclude any possible extreme values, all values above 1300 ms (1.5 T) and 2600 ms (7 T) were disregarded when estimating the average T_1_ within each ROI. In addition, the average *k* factor was calculated within IR ROIs to investigate adiabatic pulse quality.

All estimated average T_1_ values within an ROI were corrected for BMI differences between subjects with a reference BMI set to the mean value of all healthy subjects (BMI = 23.4 kg/m^2^) [24].

### Statistical analysis

Statistical analysis was conducted in Matlab (v. R2013b, Mathworks, Nattick, USA) and for all statistical testing, *P* < 0.05 was considered a significant result. Median and range were used for descriptive statistics.

To investigate a potential difference between starting at 1.5 T or 7 T, the relative T_1_ increase at 7 T was compared between the groups starting at 1.5 T and 7 T using a Mann-Whitney U test. The Pearson correlation coefficient was used to estimate the correlation between T_1_ values at 1.5 T and 7 T. The coefficient of variation, defined as the ratio of the range and median T_1_ values in the healthy and patient subject groups, was used as a measure of variability. The differences in T1 values between healthy subjects and patients were tested for the various ROIs using a Mann-Whitney U test at both 1.5 T and 7 T. The quality of the fit for IR T_1_ calculations was estimated as the standard error of the estimate (SEE) defined as the root of the averaged squared distance from the data points to the fitted line. The SEE was normalized to the estimated *S*_*0*_ and compared between using 6 and 7 TIs using a Wilcoxon signed rank test. A Wilcoxon signed rank test was also used to compare the average IR *k* factors between 1.5 T and 7 T.

The measured T1 relaxation times are expected to be longer at 7 T than at 1.5 T. To be able to compare the average T_1_ relaxation times from each ROI compartment between field strengths, the T_1_ values were normalized to the median value of all ROIs in healthy volunteers for the corresponding field strength. The resulting normalized T_1_ values were compared between 1.5 T and 7 T for each ROI using a Wilcoxon signed rank test.

Method agreement between VFA, LL, and IR were estimated using linear regression and Bland-Altman analysis. Slope, intercept, average bias, and limits of agreement were presented as measures of agreement. Inter-reader and inter-slice variability were measured as average bias and limits of agreement.

## Results

The cartilage in all included subjects were deemed of adequate thickness for ROI evaluation in deep as well as superficial regions, based on the images from the diagnostic acquisition and also directly from the images used for T1 evaluation. No cases of unexpected pathology was found.

**Fig. 1:**
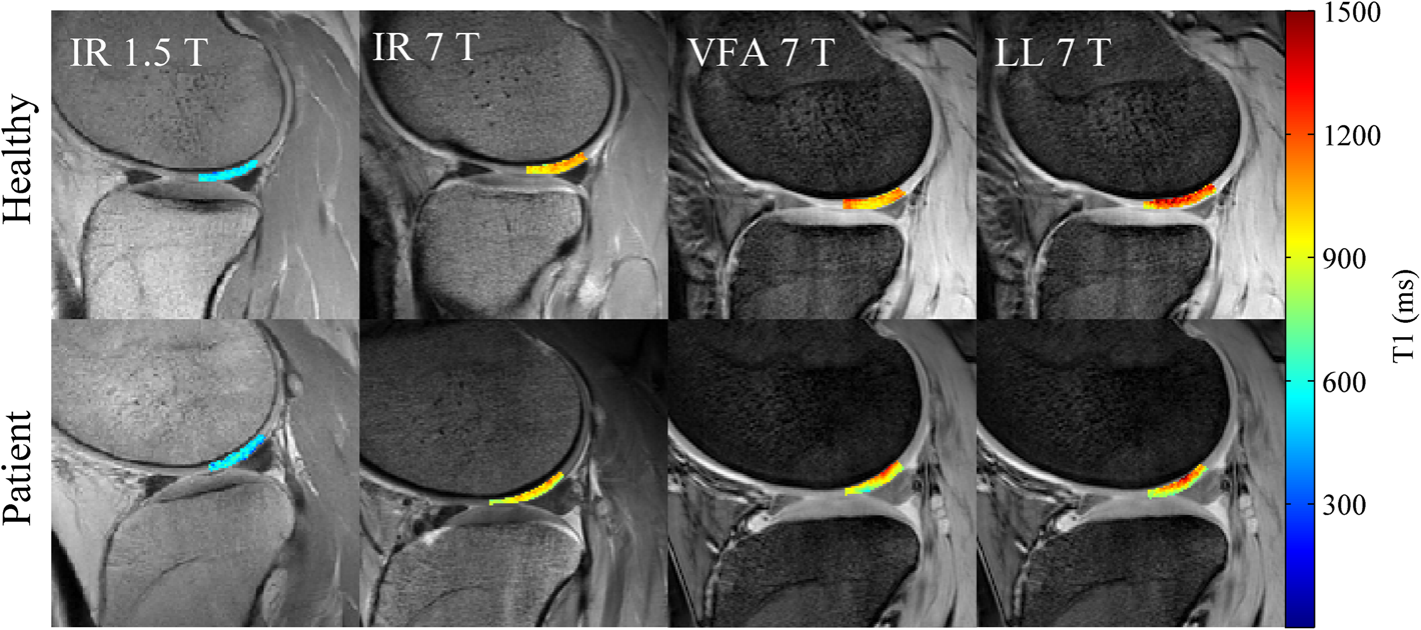
Example post-contrast T_1_ maps overlaid on raw images acquired at 1.5 T and 7 T from a healthy volunteer (top row) and a patient (bottom row). As, expected longer T_1_ values were measured at 7 T compared to 1.5 T. IR resulted in homogenous and precise T_1_ maps, whereas more variation was seen for the VFA and LL T_1_ maps

Visually, the T_1_ values within ROIs of T_1_ maps obtained with the IR sequence were regarded precise and homogenous, both from 1.5 T and 7 T (Fig. 1). In contrast, T_1_ varied considerably within the ROI for the 3D sequences (VFA and LL) in the healthy subject as well as the patient image examples.

The median T_1_ values obtained with IR at 1.5 T and 7 T for the various ROI and subject groups are presented in Table 1. As expected, longer T_1_ values were estimated at 7 T with an average factor of 1.8 (1.4–2.3) times longer 7 T T_1_ s compared to those at 1.5 T. The same overall patterns were observed at both field strengths with shorter T_1_ values in superficial compared to deep cartilage, and shorter T_1_ in the medial compared to the lateral condyle for both patients and healthy subjects. At both field strengths, patient T_1_ values were slightly shorter and with a larger spread of values compared to healthy subjects. The difference between patient T1 values and healthy subjects T1 values were however not statistically significant. In the superficial medial region the difference was close to significant both at 1.5 T (*P* = 0.11) and at 7 T (*P* = 0.09), whereas for all other ROI’s the test resulted in higher *P*-values (*P* > 0.25).

**Table 1:** Estimated T_1_ values in the femoral condyles of study subjects at the two field strengths

		T1_IR_ at 1.5 T (ms)	T1_IR_ at 7 T (ms)
Healthy (*N* = 10)			
Medial	Superficial	497 (361–562)	871 (695–1081)
	Deep	534 (459–587)	967 (854–1229)
Lateral	Superficial	530 (436–599)	944 (796–1080)
	Deep	556 (488–635)	1042 (921–1209)
Patients (*N* = 9)			
Medial	Superficial	463 (336–522)	799 (469–1031)
	Deep	507 (333–589)	984 (626–1261)
Lateral	Superficial	541 (343–624)	890 (473–1068)
	Deep	546 (364–622)	1029 (632–1193)

Values represent median (range) of the estimated T_1_

**Fig. 2:**
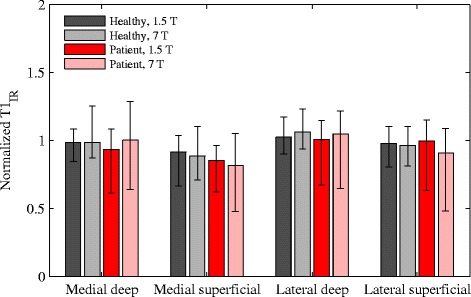
Comparison of median normalized T_1_ values at 1.5 T and 7 T in the various ROIs with error bars showing the range of values. The T_1_ values were normalized to the median T_1_ value of all healthy subject ROIs at 1.5 T and 7 T, respectively, to enable a comparison between the relaxation times at the two field strengths. Similar normalized T_1_ values was seen at the two field strengths, and a significantly larger normalized T_1_ at 7 T compared to 1.5 T was detected only in the deep lateral region in patients (*P* = 0.04)

To be able to compare the T_1_ values at the two field strengths, they were normalized to the median T_1_ in healthy subjects at each field strength (Fig. 2). The differences in normalized T_1_ values between 1.5 T and 7 T for the various ROIs were all small, with a largest relative difference of 10% in the superficial lateral region in patients. The normalized T_1_ values were not statistically different in most ROIs in neither patient (medial deep: *P* = 0.36, medial superficial: *P* = 0.16, lateral superficial: *P* = 0.13) nor healthy subjects (medial deep: *P* = 0.77, medial superficial: *P* = 0.70, lateral superficial: P = 0.70). The only exception was the deep lateral ROI were the difference in normalized T_1_ between field strengths was statistically significant in patients (*P* = 0.04) and had a low *P* value also in healthy subjects (*P* = 0.06).

The median (range) time between the medial IR sequences at the two field strengths was 40 (28–63) minutes for healthy subjects and 40 (28–49) minutes for patients. To determine if this time difference would have impact on the comparison of the dGEMRIC results between the field strengths, the ratio of T_1_ at 7 T and 1.5 T was compared between healthy subjects first scanned at 1.5 T and at 7 T, respectively. The median (range) T_1_ ratios when starting at 1.5 T / 7 T was 1.92 (1.51–2.05) / 1.67 (1.47–2.26) for the superficial ROIs and 1.82 (1.71–2.20) / 1.85 (1.72–1.99) for the deep ROIs. The difference was larger for superficial ROIs, but not statistically different for neither superficial (*P* = 0.16) nor deep ROIs (*P* = 0.62). For this reason, we do not discriminate between in which order the measurements at the two field strengths were performed in the results presented here.

**Fig. 3:**
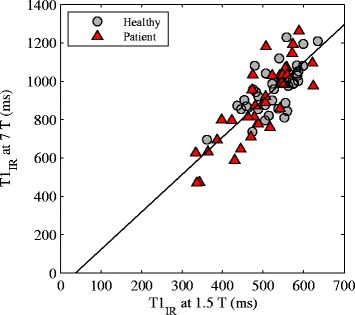
A scatter plot of IR T_1_s at 7 T against IR T_1_ at 1.5 T for healthy (grey circles) and patient subjects (red triangles) demonstrating the strong correlation between dGEMRIC at the two field strengths (Pearson correlation coefficient = 0.80). The linear regression with slope = 1.95 (1.61–2.29) and intercept = − 71.9 (− 248–104) is shown in solid black

A linear correlation was found between the T_1_ values at 1.5 T and 7 T with a Pearson correlation coefficient of 0.80 (Fig. 3). The coefficient of variation at 1.5 T/7 T T_1_ s was 0.50/0.54 for healthy subjects and 0.57/0.88 for patients, thus indicating a slightly larger spread of the 7 T T_1_ values, especially for patients. The inter-reader variability of the IR T_1_s was 5.71 ± 49.2 ms at 1.5 T and − 4.25 ± 96.1 ms at 7 T. The adiabatic pulse quality observed at 7 T was significantly lower compared to at 1.5 T with median (range) *k* factors equal to 1.85 (1.75–1.93) and 1.71 (1.11–1.89) at 1.5 and 7 T, respectively (*P* = 4·10^− 13^). Of the 38 acquired IR data sets at each field strength, three data sets at 7 T had too low SNR for a voxel-by-voxel T_1_ estimation due to a poor quality adiabatic pulse. For these data sets, an ROI-based T_1_ estimation was performed.

**Fig. 4:**
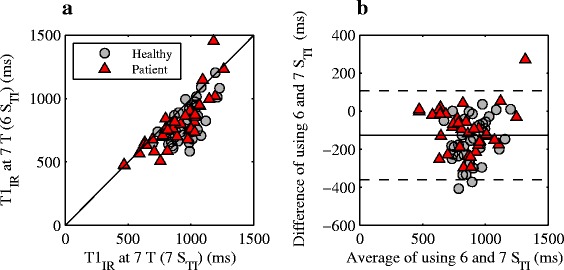
Scatter (**a**) and Bland-Altman plot (**b**) comparing IR T_1_ values at 7 T using an adapted TI sampling with an additional longer TI (7 TIs) and the IR TI sampling pattern with 6 TIs. In **a**) the line of identity is shown in solid black. In **b**) the average bias and limits of agreement are shown in solid and dashed black lines, respectively. The T_1_ values using 6 TIs were underestimated compared to using 7 TIs

Using the same six TIs at 7 T as used at 1.5 T results in lower T_1_ values (bias ± limits of agreement − 127 ± 234 ms) and a lower quality fit with significantly higher SEE (median SEE = 138 (20.9–1180) compared to SEE = 77.0 (29.4–524), *P* = 2·10^− 6^) compared to using an adapted sampling scheme with an additional longer TI at 7 T (Fig. 4). Thus, the presentation of 7 T T_1_ results is based on the adapted sampling scheme.

**Fig. 5:**
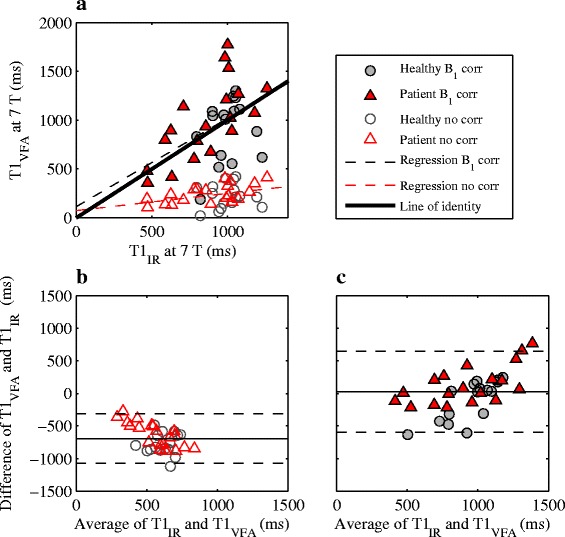
Scatter (**a**) and Bland-Altman (**b**, **c**) plots comparing the VFA and IR techniques at 7 T for healthy (grey circles) and patient subjects (red triangles). The comparison was made both for B_1_-corrected (**a**, **c**; filled markers) and B_1_-uncorrected data (**a**, **b**; empty markers). In **b**) and **c**), the average bias and limits of agreement are shown as solid and dashed lines, respectively. A poor agreement was seen between the methods, especially without B_1_ correction

A poor agreement was observed between the T_1_ values measured with VFA and IR at 7 T (Fig. 5 and Table 2). With no B_1_ correction the VFA technique severely underestimated T_1_. Although the accuracy was improved using a B_1_ correction, the precision worsened with wider limits of agreement. The inter-slice variability for the B_1_-corrected case was − 2.55 ± 433 ms and the inter-reader variability was − 51.9 ± 327 ms. Both of these estimates of variability indicate a poor precision of the VFA method. Out of the 10 acquired VFA data sets, one was excluded due to technical difficulties during imaging.

**Table 2:** Measures of method agreement between IR and the VFA and LL techniques

	VFA		LL	
	B_1_ correction	No correction	B_1_ correction	No correction
Bias ± limits of agreement (ms)	39.6 ± 640	− 687 ± 389	12.2 ± 202	−239 ± 269
Slope ± 95% CI	0.90 ± 0.57	0.18 ± 0.18	0.88 ± 0.29	0.38 ± 0.29
Intercept ±95% CI (ms)	114 ± 537	74 ± 169	119 ± 265	326 ± 274

Regression and Bland-Altman analysis was used as measures of method agreement

**Fig. 6:**
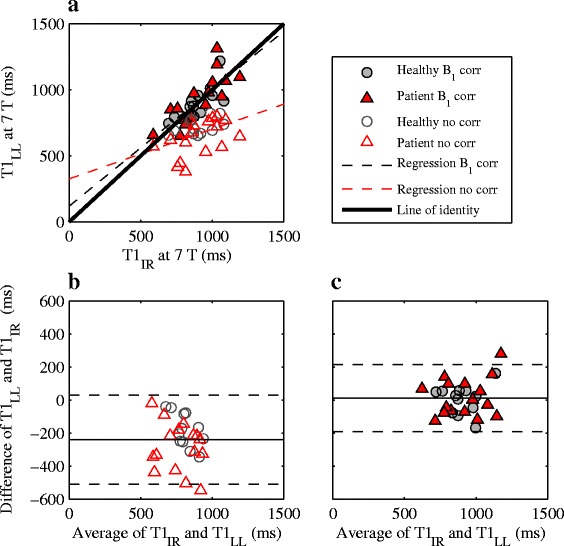
Scatter (**a**) and Bland-Altman (**b**, **c**) plots comparing the LL and IR techniques at 7 T for healthy (grey circles) and patient subjects (red triangles). The comparison was made both for B_1_-corrected (**a**, **c**; filled markers) and B_1_-uncorrected data (**a**, **b**; empty markers). In **b**) and **c**), the average bias and limits of agreement are shown as solid and dashed lines, respectively. A strong agreement was seen between the two methods, especially with B_1_ correction

The LL T_1_ values agrees well with IR T_1_ values at 7 T (Fig. 6 and Table 2). Compared to VFA, LL both with and without B_1_ correction is more accurate and precise. The agreement also for LL is improved using B_1_ correction, but the correction is not as vital as for VFA. For the B_1_-corrected LL data, the inter-slice variability was 1.33 ± 172 ms and the inter-reader variability was 11.5 ± 63.7 ms, thus indicating a smaller variability of LL data compared to VFA. Out of the 14 acquired LL data sets, five were excluded due to insufficient B_0_ shim causing a failed adiabatic inversion pulse, and two were excluded due to technical difficulties during imaging.

## Discussion

This study compared dGEMRIC at 1.5 T and 7 T after a single (one) contrast-agent injection in both healthy subjects and patients. A randomized alternation of the 1.5 T and 7 T examinations of healthy subjects indicated that it was possible to obtain both measurements after a single contrast-agent administration without significant bias due to the difference in time delay after injection. Using an adapted sampling scheme with an additional longer TI, dGEMRIC based on T_1_ measurements with IR is feasible at 7 T. The estimated T_1_ values at the two field strengths were strongly correlated, although there was a slightly wider distribution of the 7 T T_1_ s. Similar normalized T_1_ values were found using the two field strengths with significant, yet small, difference between the two only for the deep lateral region. Out of the investigated 3D options at 7 T, LL showed a higher agreement to IR results compared to VFA. For both LL and VFA, B_1_ correction is necessary at 7 T. Careful B_0_ shimming is crucial, especially for the IR and LL methods.

The feasibility of dGEMRIC at 7 T has previously been studied for knee [16] and hip applications [17, 18]. Our estimated postcontrast femoral T_1_ values are slightly longer than those presented by Welsh et al. in healthy volunteers [16]. In expectation of longer T_1_ values at 7 T, we chose to use an additional longer TI, compared to what was used in the Welsh study. The results from our study indicate that this choice is necessary to avoid underestimation of T_1_ at 7 T.

dGEMRIC has previously been compared between 7 T and a clinical field strength in repaired cartilage tissue of the hip, where dGEMRIC at 7 T resulted in an unexpected T_1_ decrease compared to dGEMRIC at 3 T [18]. Our study, however, resulted in the expected markedly longer T_1_ values at 7 T compared to 1.5 T in native knee cartilage of both healthy volunteers and patients. In the previous study [18], the VFA technique was used, while we in contrast chose an IR T_1_-mapping method for the field strength comparison. Based on the evaluation of these techniques at 7 T presented in our current study, we believe that this resulted in a more accurate comparison of T_1_ values between field strengths.

VFA, LL, and IR have previously been compared at clinical field strengths [25]. Similarly to the results in this study, both the accuracy and precision of LL was superior to VFA also at 1.5 T. Previous studies using dGEMRIC at 7 T has used IR [16] and VFA [17, 18] for T_1_ mapping. In a few healthy hips, these two techniques have also been semi-quantitatively compared at 7 T and their respective measures were considered comparable [17]. Success of the VFA technique is dependent on an optimal choice of flip angle pair and B_1_-inhomogeneity correction at high field strengths [11, 15]. Thus, at 7 T VFA is expected to be especially challenging as B_1_ inhomogeneity is likely high enough to also affect the optimal choice of flip angles. This issue may explain why VFA showed poor agreement with IR even after B_1_ correction in our quantitative comparison.

The inversion pulses used in IR sequences were of better quality at 1.5 T compared to at 7 T in this study. This might be explained by the use of a transmit/receive coil at 7 T compared to a receive-only coil at 1.5 T. In addition, the adiabatic-type pulses rely on a successful B_0_ shim which is more challenging at 7 T. Especially, this issue was apparent for the LL technique of which several data sets had to be excluded for this reason. However, also a few IR data sets suffered from poor SNR due to this problem. The B_0_-shim procedure was improved during the course of the study, and after the volume-based first-order shim technique first used was replaced by use of the Shimtool [26] (an image-based second-order shim technique) the shim was sufficient in all the remaining LL data sets and the SNR of the remaining IR data sets were consistently high.

In our implementation, dGEMRIC using IR required a longer scan time at 7 T compared to at 1.5 T. The reason is that we, as we expected longer T_1_s at the higher field strength, chose to increase the repetition time and add an extra acquisition with longer TI at 7 T. Our comparison of using six and seven TIs for the T_1_ estimation demonstrates that this was necessary to achieve an accurate T_1_ estimation at 7 T. However, we also noticed that the longer acquisition time made the sequences more sensitive to patient motion as motion correction was more frequently needed in the 7 T scans as compared to the shorter 1.5 T scans. In a practical case when designing a study protocol it is important to take both the benefits and the potential disadvantages of a longer scan time into consideration.

Focus of this work was on the T_1_-mapping techniques used for the dGEMRIC method at 7 T, and patients were primarily recruited to increase the expected range of T_1_ values. For this reason, a full comparison of dGEMRIC indices and diagnostic performance was beyond the scope of this study. However, although the difference in post-contrast T_1_ values between patients and healthy volunteers was small (not statistically significant) in this study, it was similarly small at both 1.5 T and 7 T. At both field strengths it was the same region (superficial medial) that was closest to a significant difference, which is also the region were cartilage changes had been observed in the patients. The normalized T_1_ values were also similar at the two field strengths. This hence implies that the methods perform similarly at the two field strengths. As possible explanation for the small differences found, we speculate that the difference in timing between the protocols starting at 1.5 T and 7 T may have increased the spread of the data making comparisons between groups more difficult.

dGEMRIC using Gd-(DTPA)^2-^ (Magnevist) will probably be performed less frequently in the future given the fact that its use will be restricted based on the recent reports about long-term gadolinium deposits [5]. However, dGEMRIC could potentially also be used with other contrast agents such as gadoterate meglumine (Dotarem, Guerbet, Villepinte, France). To date almost all dGEMRIC studies have been performed using Magnevist, and future dGEMRIC studies with other contrast agents would of course first need careful and in depth validation studies. After such validation, the results from our evaluation of T1 mapping methods and field strength comparison would still be valuable for future studies with dGEMRIC at 7 T.

There were mainly two limitations of the study design of this work: the time difference between the examinations at the two field strengths and that no precontrast T_1_ values were measured. Both for ethical and study design purposes, the examinations at the two field strengths were conducted after a single (one) contrast agent injection. Thus they could not be performed at identical post-injection time delays. Efforts were made to minimize the time delay between examinations and the subjects were moved in a wheel chair between the examination rooms to avoid loading the knee and thus redistributing the contrast agent between measurements. The achieved time delay between examinations are within the previously reported plateau of dGEMRIC values between 2 h and 3 h after injection [27]. Although no statistically significant difference in T_1_ was found due to timing differences in healthy volunteers, they may have increased the spread of the data as mentioned in the paragraph above. Precontrast T_1_ values were not measured neither at 1.5 T nor 7 T. This choice was made as the addition of these measurements would make the visit and scan time unbearably long for the study subjects. Instead, it was prioritized to make it feasible to perform the examinations at the two field strengths in a single visit and after a single contrast agent injection. Previous work indicates that the precontrast T_1_ value contributes little additional information compared to postcontrast values at both 1.5 T and 3 T in native cartilage [3, 28]. In repaired cartilage tissue, measurements of precontrast T_1_ may be more important [29]. The importance of a precontrast T_1_ value may need to be investigated further also at 7 T.

## Conclusions

In conclusion, T_1_ mapping for use in the dGEMRIC method is feasible at 7 T with similar normalized T_1_ values compared to at 1.5 T and with a strong correlation between T_1_ values at 1.5 T and 7 T. However, the IR protocol at 7 T needs to be adapted to the longer T_1_ values at this field strength. As a 3D alternative to IR at 7 T, LL is preferred and VFA is not recommended without further optimization of the method. For both 3D methods, B_1_ correction is necessary for an accurate T_1_ estimation. For LL and IR, careful B_0_ shimming is crucial at 7 T.

## Abbreviations

BMI: Body mass index
CEST: Chemical exchange saturation transfer
dGEMRIC: Delayed gadolinium enhanced magnetic resonance imaging of cartilage
DREAM: Dual refocusing echo acquisition mode
FOV: Field of view
GAG: Glycosaminoglycan
IR: Inversion recovery
LL: Look-locker
MRI: Magnetic resonance imaging
ROI: Region of interest
SEE: Standard error of the estimate
TE: Echo time
TI: Inversion time
TR: Repetition time
VFA: Variable flip angle
